# Case of Chronic Mortification

**Published:** 1856-12

**Authors:** 


### CASE OF CHRONIC MORTIFICATION.

BY D. C. D.

Messrs. Editors: I subjoin some imperfect notes of a case which came under my notice awhile since. As it is a case which considerably interested me at the time it was under observation, I am induced to believe that a recapitulation of some of its prominent points, will not now prove altogether uninteresting or unacceptable to your readers. Having neglected during its progress to make memoranda for future reference, I have to rely upon memory, and my statements, as far as dates, &c., are concerned, must be considered only as approximations to exactness.

The patient, in question, Mr. F., a resident of the State of N. York, and aged about 74 years, had for many years enjoyed tolerable health, though he had experienced some trouble from an erysipelatous affection, so called, which had shown itself mostly in his eyes. His habits at this date, were entirely temperate, though he had been, many years previously, as I learned from his friends, a hard drinker.

I was called to see the patient about Oct. 1st., 1855, on account of a difficulty with one of his great toes, which caused him much inconvenience, and which I found presenting the appearance of an abrasion upon the upper surface and near the articulation of the first and second phalanges.

This difficulty was attributed by him to the wearing of an ill-fitting shoe. There was also an appearance of erysipelatous inflammation in the adjacent portions of the foot, shown by some redness and swelling, as well as increased heat. The inflammatory condition of the eyes, it may be noted, had disappeared.

Thinking nothing further indicated at the time, I made use merely of mild local applications, to relieve the irritability and excitement of the part, and promote the healing of the excoriation, conjoining the use internally of gentle aperients. The healing process, however, was not forwarded by these measures. The boundaries of the excoriation were very slowly extended, and the toe assumed gradually a livid hue, losing by slow degrees its vitality, and approximating, together with the neighboring parts, a cadaveric coldness. As this condition of things made its appearance, a more stimulating treatment seemed to be called for, and was pursued. In addition to other measures, a narrow blister was laid across the foot near the toes, but still the disease moved on, steadily, though slowly.

At this time, say Nov. 1st, it was deemed advisable that a consultation should be had in reference to the case, and Dr. F., of Utica, a highly respectable practitioner, and formerly the family physician, was called upon. In consultation, it was determined that a tonic course should be pursued. Quin. sulph. 1 gr. three times daily, nourishing diet, &c., were directed. To the toe we applied mild stimulants: ung zinc, &c.; the discharge from the sore at this time was not profuse, and was of an ichorous quality. For keeping up warmth and circulation in the foot, wrapping it thickly with carded wool was suggested by Dr. T., and was resorted to, apparently with some effect. The loss of vitality, however, proceeded subsequently, and with greater rapidity, and the whole thickness of the lower portion of the toe became completely dead and blackened, as well as desicated. From some portions an offensive matter exuded; to correct which, I applied liq. sod. chlor., dilute.

Recollecting at this time a statement by one of my old medical teachers, Prof. Lee, of New York, regarding the alledged efficacy of sulpuric acid, in this form of disease, I determined to try it, and accordingly gave ten drops of the strong acid three times daily. With the apparent effects of this, I was gratified. The edges of the living parts very soon assumed a more healthy and florid hue; the secretions approached more nearly the character of healthy pus, and a more distinct separation of the dead from the living parts occurred.

While I would not confidently assume for the acid all the credit of this marked improvement, and while I am aware that accidental coincidences and sequences are often mistaken for positive results, I am yet disposed to allow to it an important agency in the present case.

Nature now seemed to be making good progress towards an amputation. The soft parts above became completely separated, also a portion of the bone. and I was led to doubt somewhat the propriety of any mechanical interference which should supersede her operations.

Upon a subsequent consultation, however, with Dr. T., I acceded to his desire to remove the toe at once. Upon the head of the metataesal bone being sawed through, it was found to be somewhat softened. But very slight hemorrhage resulted from the wounding of the soft parts. The amputation was performed with the patient under the influence of chloroform, and he was not afterwards sensible of having suffered pain. The wound was dressed in the ordinary manner with adhesive straps. Cicatuzation took place very slowly, requiring, perhaps, two months for its entire completion. Considerable pus was, meanwhile, secreted, and for a time it was of a somewhat fœted character. Spasmodic action of the muscles of the foot and leg, which had, during the previous history of the case, caused some inconvenience, was henceforth aggravated. Continuing during the entire period of cicatuzation, and for sometime subsequently, it was often productive of intense suffering. The patient was under the necessity of taking anodynes habitually, and sometimes in very large doses. Permanent contraction of the flexer muscles also took place, so that the leg hung at a considerable angle with the thigh. An additional difficulty now became complicated which was a source of much discomfort; this was an eruption affecting the feet and legs, and finally, to a certain degree, the whole body. As it first appeared I considered it to have been eczematous in its character, and it was attended with excessive pruritus, and a copious honey-like discharge. It took on at length, a squamous form, and after having been assailed with the various remedies, finally yielded to the administration of arsenic, in the form of Fowler's solution, and the local application of black wash. It should be added, that the tonic measures were continued during nearly all the latter period of treatment. Iron was used in addition to the quinia, also, beer and brandy to a certain extent.

This disease is one, which, since the days of Polt, has attracted considerable attention from the profession, and considerable progress has, no doubt, been made, towards just views of its pathology and principles of treatment. Much, however, evidently yet remains to be done, and considering its not unfrequent occurrence, and its hitherto fatal results, it behoves every practitioner who has opportunities for observation, to endeavor to prosecute his investigations in such a manner as to shed some light on the subject, and to add something to that stock of facts, upon which, alone, must be based all rational and successful treatment.

Chronic Mortification can hardly be considered a disease, *per se*, but should rather be looked upon as a sympton, which may result from a variety of conditions and predisposing circumstances. It occurs commonly in old persons and in wornout constitutions, though this is, by no means, universally the case. It occasionally makes its appearance in young and, apparently, healthy subjects; and we may, perhaps, be able, in some of these cases, to satisfy ourselves that it is the result of undue excitement of a part, rather than of depressed action, and that it requires the antiphlogistic treatment as recommended by Baron Dupuytren, and practiced by Gibson in an instance which he relates.

Doubtless no exclusive or empirical treatment can be relied upon. The physician must study closely the condition and attending circumstances of each individual case. If enfeebled power of the heart, and depression of the vital actions, generally, from whatever cause produced, exist as predisposing causes, they must be met promptly and energetically by tonics and stimulants; at the same time any more immediate exciting cause which may be in operation, should be looked after and removed, or counteracted as far as possible. If arteritis or capillery inflammation be diagnosed as the cause of the trouble, we have, of course, an entirely different class of remedies indicated, viz: the antiphlogistics. If old age, with its ossific deposits in the vessels, its feeble constitutional power, and poverty of blood, as regards both quantity and quality, be the co-existing circumstances, we must regulate our treatment with reference to these different conditions. In one case a certain principle of treatment should be regarded, in another, perhaps, an entirely different one, while a third, may require a continuation of the two methods.

The various cases of Chronic Mortification have been arranged by some writers, under the head of wet and dry. This, I consider, a somewhat arbitrary division, and unsatisfactory as regards indications of pathology or treatment; though, of course, as regards pathology, dryness would lead us to infer an obstruction to the arterial circulation. I believe, too, it will be found in practice, that the wet and dry conditions in different cases run into each other by successive gradations.

In the case of which I have given a partial history, very considerable ossification of the arterical system was evident. Probably there was also a deteriorated quality of the blood, and to this was added a certain degree of constitutional feebleness. The irritation and abrasion of the toe may be looked upon as an immediate exciting cause of the mortification; the other circumstances mentioned, standing somewhat in the position of predisposing causes.

The action of the sulph. acid, which seemed so highly efficient, I am unable to define with precision; in addition to its tonic influence, I am disposed to attribute to it an illy understood alterative action upon the blood, by virtue of which it perhaps eliminates from it the effete and decaying particles bearing this fluid in a more healthful and limpid condition, with greater capacity for permeating the minute and contracted vessels of distant parts, and for promoting healthful assimilation and secretions.

Should I meet with another case similar to the one described, I should be inclined to give the sulph. acid an early trial; I would add to this other sustaining measures, and, perhaps, conjoin the use of cod liver oil. Locally, I should aim to keep up the natural warmth, perhaps, varying somewhat my applications to meet the varying sensible conditions of the part.

KEOKUK, Dec. 8, 1856. D. C. D.

